# Analysis of the Diagnosis of Burkitt-Like Lymphoma in a Patient With Atypical Cytogenetics and Molecular Markers

**DOI:** 10.7759/cureus.28295

**Published:** 2022-08-23

**Authors:** Jackson R Brunner, Ellery Altshuler, Li-Jun Yang

**Affiliations:** 1 Department of Internal Medicine, University of Florida College of Medicine, Gainesville, USA; 2 Department of Pathology, Immunology, and Laboratory Medicine, University of Florida College of Medicine, Gainesville, USA

**Keywords:** high grade neoplasm, diagnostic algorithm, world health organization, high-grade b-cell lymphoma, diffuse large b-cell lymphoma

## Abstract

The World Health Organization (WHO) criteria for diagnosis of hematopoietic and lymphoid cancers serve as a useful t­ool for distinguishing between malignant conditions based on phenotypic, morphologic, and/or cytogenetic presentations, but their utility is limited in patients whose diseases contain elements of multiple diagnoses. We present a case of a 59-year-old male with enlargement of muscular and soft tissues of the left hip and an intraconal soft tissue mass surrounding the left optic nerve, who was treated for Burkitt-like lymphoma (BLL). Cytogenetics revealed the absence of an MYC rearrangement involving chromosomes 2, 14, or 22, normally found in Burkitt lymphoma, or the classic telomeric losses and proximal gains observed in BLL. Diffuse large B-cell lymphoma, not otherwise specified (DLBCL, NOS) and high-grade B-cell lymphoma, not otherwise specified (HGBL, NOS) were also considered as possible diagnoses. The persistence of ambiguous lymphoma diagnoses demonstrates the need for both continued research in the area and regular revision of the WHO criteria. Physicians working with patients with poorly defined lymphomas should defer to diagnostic algorithms where applicable, many of which have been proposed in the literature.

## Introduction

Burkitt lymphoma (BL) is an aggressive B-cell malignancy primarily affecting children and young adults (both endemic and sporadic types) and often associated with prior Epstein-Barr virus (EBV) infection. The disease is classically associated with a translocation of the MYC gene on chromosome 8 with genetic material from chromosomes 2, 14, or 22 [1]. This classification is one of many delineated by the WHO in its guidelines for the diagnosis of hematopoietic neoplasms, the latest edition of which was released in 2016 [2]. These categories often share morphologic, cytogenetic, and clinical features with one another, which can complicate accurate diagnosis.

## Case presentation

Our patient was a 59-year-old white male without significant past medical history who presented with a two-month history of worsening left hip and gluteal pain radiating down the leg. He also complained of blurry vision that began around the time the pain had started. When the pain became unbearable, he presented to the emergency department where a complete blood count (CBC) showed the following: white blood count 7.8 thousand/mm3 (reference: 4-10 thousand/mm3) with 43% neutrophils (reference: 40-80%), 44% lymphocytes (reference: 20-45%), and 8% abnormal immature cells-blasts atypical lymphocytes, hemoglobin 11 grams/dL (reference: 13.0 - 16.5 grams/dL), hematocrit 34.6% (reference: 40 - 50%), and platelets 19,000 thousand/mm3 (reference: 150-450 thousand/mm3). Lactate dehydrogenase was 1800 units/liter (reference: 135-225 U/L). EBV serology was negative. He denied fevers, chills, night sweats, weight loss, chest pain, shortness of breath, nausea, vomiting, or abdominal pain. The patient had no family history of cancer and had never smoked cigarettes.

**Figure 1:**
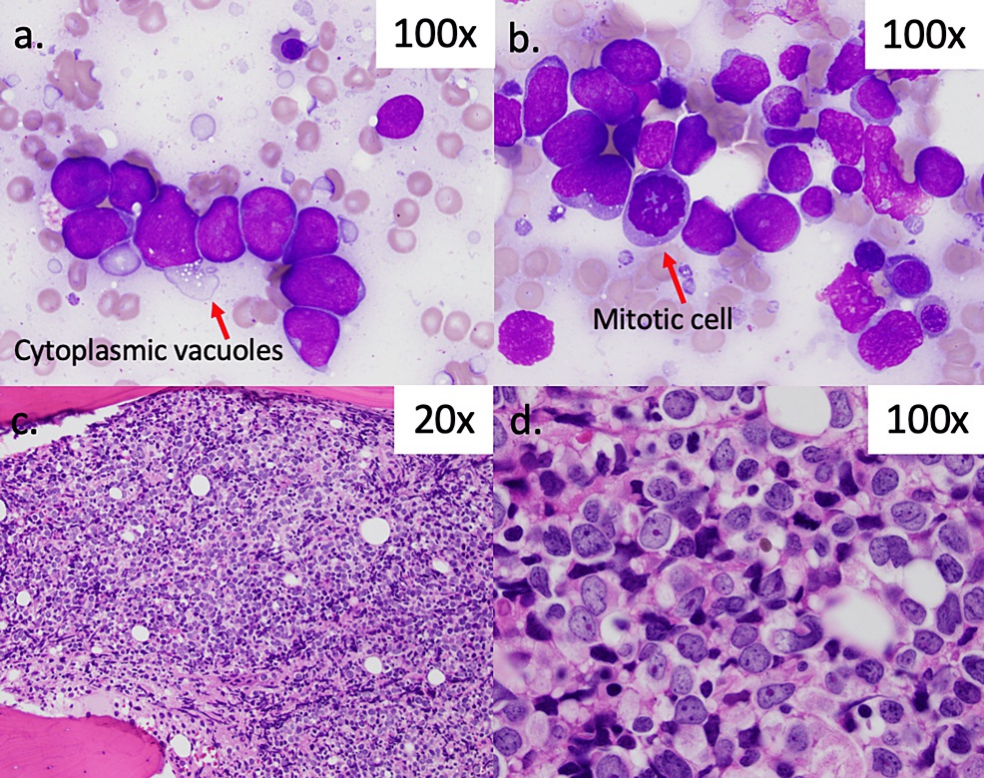
Bone Marrow Aspirate and Biopsy. Bone marrow touch prep (a/b) showed many abnormal, large lymphoid cells displaying enlarged nuclei and often multiple nucleoli. Cytoplasmic vacuoles were observed in some of these cells. Bone marrow biopsy (c/d) revealed significant hypercellularity and prominence of large cells with atypical nuclei, dark nucleoli, and irregular nuclear membranes.

The bone marrow aspirate and biopsy findings are summarized in Figure 1.

**Figure 2:**
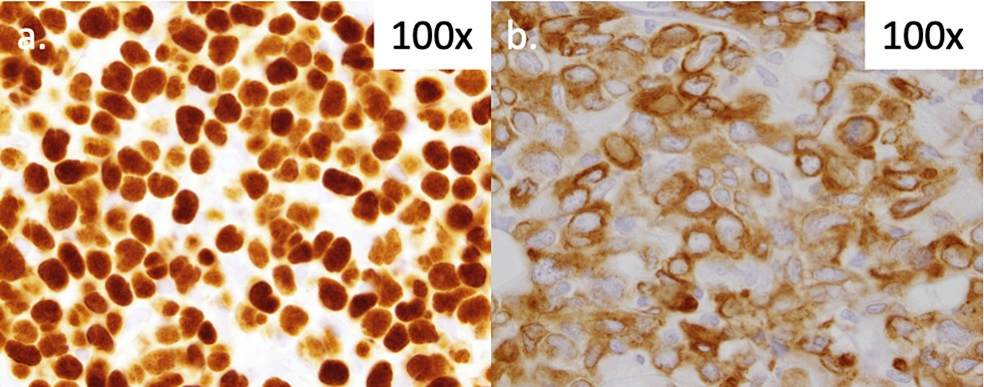
Immunohistochemistry. Staining demonstrated PAX5 (a) and CD79a (b) positivity.

The aspirate was 80-90% cellular with 90% involvement by high-grade B cell leukemia/lymphoma. There was co-expression of cluster of differentiate (CD)19, CD10, and terminal deoxynucleotidyl transferase (TdT) with kappa-surface light chain restriction and variable CD20 and CD5. Immunohistochemical (IHC) studies were positive for CD79a, TdT, and PAX5 (Figure 2) and negative for BCL6, cyclin-D1, and SOX11. Ki-67 expression was high. Cytogenetics showed complex tetraploidy with add(2), add(3), add(4), add(8), and add(9). Fluorescence in situ hybridization (FISH) was positive for copy number gain for all loci, and for MYC gene locus rearrangement (not IgH/MYC). FISH was negative for BCL6, BCR/ABL, BCL2, and IgH rearrangements. 

Lumbar puncture was performed, and cerebrospinal fluid (CSF) was negative for disease involvement. The cancer was considered stage IVA and high-grade. There was significant debate about how to classify the malignancy; eventually, it was called B-cell Burkitt-like lymphoma (BLL) that had presented in the leukemic phase. (Note that his cancer was diagnosed prior to the 2016 revision of the WHO criteria, and thus the label “Burkitt-like lymphoma” the disease was given did not indicate the specific chromosomal changes highlighted therein.)

Treatment was initiated with rituximab and hyperfractionated cyclophosphamide, vincristine, sulfate, doxorubicin hydrochloride, and dexamethasone (R-hyperCVAD) with intrathecal cytarabine and both systemic and intrathecal methotrexate. After three cycles of chemotherapy, the patient developed a severe headache and was found to have disease progression into the leptomeningeal space. The patient received radiation treatment and intrathecal methotrexate and chemotherapy. One cycle of rituximab, ifosfamide, carboplatin, and etoposide was administered; however, the chemotherapy was not well tolerated, and his functional status significantly worsened. He elected to pursue hospice care and died 13 months after symptom-onset and 11 months after beginning treatment. 

## Discussion

Our case posed a diagnostic challenge as the tumor did not fit neatly within the WHO criteria for hematopoietic and lymphoid cancers. Among the possible diagnoses considered were B-cell acute lymphoblastic leukemia/lymphoma (B-ALL) and mature B-cell lymphomas including BL, diffuse large B‑cell lymphoma, not otherwise specified (DLBCL, NOS), and high-grade B-cell lymphoma, not otherwise specified (HGBL, NOS). As the treatment regimen for this highly aggressive tumor depended on classification, each of these diagnoses was given careful consideration.

This patient’s CBC revealed a large increase in lymphocytes compared to the total white blood cell count. Such relative lymphocytosis is consistent with a lymphoproliferative neoplasm such as chronic lymphocytic leukemia, B-ALL, or non-Hodgkin lymphoma; however, it is also associated with a number of infections (particularly EBV), asplenia, and even stress [3]. The tumor cells histologically most closely resembled BL. The diagnosis of BL is made based on a combination of findings rather than any single parameter. These findings include any MYC gene translocations and high expression of B-cell antigens (CD19, CD20, CD22, CD79a, and PAX5), and germinal center markers (CD10 and BCL6) in the absence of BCL2 expression. The translocation of the MYC gene (8q24) to any of three places is considered the molecular hallmark of BL [2]. While MYC rearrangements can occur in other forms of lymphoma (they are present in about 10% of DLBCL, for instance) [4], they are always present in BL [5]. Eighty percent of translocations involve the Ig heavy chain on chromosome 14, t(8;14), while about 15% involve the kappa light chain on chromosome 2, t(2;8), and 5% involve the lambda light chain on chromosome 22 [6,7]. Our patient had an MYC break-apart, indicating that an MYC rearrangement was present. However, our patient did not have rearrangements involving chromosomes 2, 14, or 22. 

It was considered unlikely that our patient had BL given the absence of a classic MYC rearrangement, discordance among other cytogenetic markers like BCL6, and the presence of complex tetraploidy, which would be highly unusual for BL (usually diploid). The tumor also showed TdT positivity, which is rare in this type of cancer and more common in B-ALL. Given our patient’s low abnormal blast count (<8%), kappa-surface light chain restriction, and large cells on histology, a mature B-cell neoplasm would be a more appropriate diagnosis than B-ALL. It is unusual, however, that such a lymphoma would express immature B-cell markers, and thus this case likely represents a rare instance in which a mature B-cell neoplasm acquired immature cell markers late in the disease course, few cases of which have been reported in the literature [8]. 

Another consideration was that the malignancy should be classified as either DLBCL, NOS or HGBL, NOS. DLBCL, NOS is defined based on nuclei more than twice normal size and incompatibility with the definitions of any other DLBCL subtype. Like DLBCL, NOS, HGBL, NOS is a diagnosis of exclusion. (It has replaced the 2008 WHO diagnosis of B-cell lymphoma, unclassifiable, with features intermediate between DLBCL and BL). HGBL, NOS cannot have both an MYC rearrangement and either BCL2 or BCL6 rearrangements and presents with proliferation of blast-like cells [2]. Our patient's cancer did have an MYC rearrangement but not BCL2 or BCL6 rearrangements, and cells displayed both mature and immature markers, making HGBL, NOS a possible diagnosis. 

Algorithms have been proposed to help adjudicate between the diagnoses proposed in the 2016 revision of the WHO criteria. For example, the checklist published by King and Lam (2019) would suggest DLBCL, NOS as an appropriate diagnosis for our patient, given that his cancer was a cyclin-D1-, CD10+, BCL6-, lymphoid, large B-cell lesion with a high labeling of Ki-67 [9]. While these diagnostic tools are useful, their utility is limited in cases like ours in which the diagnosis is ambiguous, and a different conclusion can be reached depending on which elements of the case are emphasized. Ultimately, the patient in our case was treated as if he had BLL. More research is needed to optimize the treatment of cases with ambiguous lymphoma diagnoses.

There are also several published algorithms used to predict the prognosis of patients with DLCBL, NOS. One of the earliest and most widely recognized of those published, the Hans algorithm, divides cases of DLBCL into germinal center lymphomas and non-germinal center lymphomas (further divided into activated B-cell lymphoma and lymphoma with type III gene expression) based on the expression of CD10, BCL-6, and MUM1 [10]. Assuming DLBCL, NOS was the appropriate diagnosis, our patient’s disease, which expressed CD10, would be considered a germinal center lymphoma, which is generally a more favorable diagnosis [10]. 

As the classifications are relatively new, there has been little data published on appropriate chemotherapy regimens for DLBCL, NOS and HGBL, NOS. Overall survival in patients with either diagnosis is poor, especially in those treated with traditional R-CHOP (rituximab, cyclophosphamide, doxorubicin, vincristine, and prednisone). It has been suggested that dose-adjusted-EPOCH (etoposide, prednisone, vincristine, cyclophosphamide, and doxorubicin) with rituximab be used due to its ability to treat both DLBCL and BL [11]. If our patient’s disease, initially treated with R-hyperCVAD therapy with CNS prophylaxis, was indeed DLBCL, NOS or HGBL, NOS, he likely would have benefited from an alternative regimen such as that described above. 

## Conclusions

Despite regular updating of the WHO guidelines for the classification of hematopoietic and lymphoid cancers, lymphomas of uncertain subtypes such as that reported here endure. Accurate diagnosis of these cases requires a thorough review of a patient’s clinical presentation, cytogenetics, and histology. Interdisciplinary collaboration and a thorough review of the literature are crucial as these diagnostic criteria are continually being refined. Further research into appropriate treatment regimens for these cancers is warranted. Physicians should strive to engage patients with ambiguous lymphoma diagnoses in honest, informative conversations regarding the nature of their disease.
